# Fast Joint Multi-Robot Trajectory Optimization by GPU Accelerated Batch Solution of Distributed Sub-Problems

**DOI:** 10.3389/frobt.2022.890385

**Published:** 2022-07-08

**Authors:** Dipanwita Guhathakurta, Fatemeh Rastgar, M. Aditya Sharma, K. Madhava Krishna, Arun Kumar Singh

**Affiliations:** ^1^ International Institute of Information Technology, Hyderabad, India; ^2^ Institute of Science and Technology, University of Tartu, Tartu, Estonia

**Keywords:** multi-robot trajectory optimization, batch optimization, convex, collision avoidance, obstacle avoidance, GPU accelerated optimizer

## Abstract

We present a joint multi-robot trajectory optimizer that can compute trajectories for tens of robots in aerial swarms within a small fraction of a second. The computational efficiency of our approach is built on breaking the per-iteration computation of the joint optimization into smaller, decoupled sub-problems and solving them in parallel through a custom batch optimizer. We show that each of the sub-problems can be reformulated to have a special Quadratic Programming structure, wherein the matrices are shared across all the problems and only the associated vector varies. As result, the batch solution update rule reduces to computing just large matrix vector products which can be trivially accelerated using GPUs. We validate our optimizer’s performance in difficult benchmark scenarios and compare it against existing state-of-the-art approaches. We demonstrate remarkable improvements in computation time its scaling with respect to the number of robots. Moreover, we also perform better in trajectory quality as measured by smoothness and arc-length metrics.

## 1 Introduction

Deployment of multiple aerial vehicles such as quadrotors is critical for applications like search and rescue and exploration and mapping of large areas (Schranz et al., 2020). Over the last decade, robot fleets have also become ubiquitous in applications like ware-house automation that have a substantial economic impact on society (Li et al., 2020; Bolu and Korçak, 2021). Furthermore, with the advent of connected autonomous cars, it becomes imperative also to view urban mobility as a multi-robot system (Zhou et al., 2017). A fundamental component of any multi-robot system is the coordination planning that guides individual robots between their start and goal locations while avoiding collisions with the environment and other robots. In this paper, we adopt the optimization perspective for multi-robot motion planning (Rastgar et al., 2021). In this context, the existing approaches broadly fall into two spectra. On one end, we have the centralized approaches wherein the trajectory of all the robots are computed together. The centralized approach can be further subdivided into sequential (Chen et al., 2015), Park et al. (2020) and joint optimization (Augugliaro et al., 2012; Rastgar et al., 2021) respectively depending on whether the trajectories of the robots are computed one at a time or simultaneously. On the other end of the spectrum, we have online distributed model predictive control (DMPC) (Luis et al., 2020; Soria et al., 2021) based approaches wherein each individual robot computes its trajectories in a decoupled manner based on the trajectory prediction of the other robots in the environment. In some works, the prediction module is replaced by robots communicating their current trajectory with each other (Luis and Schoellig, 2019).

Centralized approaches, especially the joint optimization variants, provide a rigorous treatment of the collision avoidance constraints and access a larger feasible space formed by all trajectory variables of all the robots. However, joint optimization quickly becomes intractable as the number of robots increases (Chen et al., 2015). In contrast, the distributed MPC approaches can run in real-time but can lead to oscillatory behaviors, and consequently, low success rates of collision avoidance (Luis and Schoellig, 2019; Luis et al., 2020). This is because the trajectories computed at each control cycle by any robot are only collision-free with respect to the predicted (or prior communicated) trajectories of other robots and not the actual trajectories followed by them.

Our main motivation in this paper is to improve the computational tractability of multi-robot trajectory planning using a distributed optimization approach to the extent that it becomes possible to compute trajectories for tens of robots in densely cluttered environments in a few tens of milliseconds. To put in context, the said timing is several orders of magnitude faster than some of the existing approaches for joint multi-robot trajectory optimization (Augugliaro et al., 2012; Bento et al., 2013). Such improvements in computation time would ensure the applicability of our approach for even online re-planning besides the standard use case of computing offline global trajectories for the robots. For example, consider a scenario wherein each robot uses local real-time planners such as Dynamic Window Approach (Fox et al., 1997) or DMPC (Luis et al., 2020) to avoid collisions with other robots in a distributive manner. Our approach could provide global re-planning for the local planners at more 5 Hz. or more.

On the application side, our main focus is on coordination of multiple quadrotors, typically for applications like search and rescue and coordinated exploration. These applications require point-to-point, collision-free navigation and forms the main benchmark in our experiments. However, our algorithm can be useful for coordination of multiple wheeled mobile robots and even autonomous cars.**Contributions:** The computational efficiency of our approach is built on several layers of reformulation of the underlying numerical aspects of the joint multi-robot trajectory optimization. We summarize the key points and the benefits that it leads to below.**Algorithmic:** Our main idea is to break the per-iteration computation of the joint multi-robot trajectory optimization into smaller, distributed sub-problems by leveraging the solution computed in the previous iterations. Although similar ideas have appeared in many existing works (Bento et al., 2013; Luis and Schoellig, 2019), a core challenge remains: how to efficiently solve the decoupled problem arising at each iteration in parallel. The basic assumption is that the decoupled optimizations can be parallelized across separate CPU threads (Bento et al., 2013). However, our recent works have shown that such a parallelization approach does not scale well with an increase in the number of problems (Adajania et al., 2022). The inherent limitation stems from the available CPU cores and thread synchronization issues.

Thus our main algorithmic contribution in this paper lies in deriving a novel optimizer that can be efficiently run in a batch setting. In other words, our optimizer can take a set of decoupled optimization problems and vectorize the underlying numerical computations across multiple problem instances. Consider an optimizer that solves a given problem by adding two vectors as a hypothetical example. We can trivially vectorize the computation over different problem instances by stacking each problem’s vectors together in the form of a matrix and adding them together. Moreover, this matrix addition can be easily parallelized over GPUs for many problem instances. Our proposed optimizer achieves similar vectorization but for a set of difficult non-convex sub-problems, resulting in each iteration of joint multi-robot trajectory optimization. Specifically, we show that solving the decoupled sub-problems predominantly reduces to solving novel equality constrained quadratic programming (QP) problems under certain collision constraint reformulations. The novelty of the QPs stems from the fact that they all share the same matrices (e.g., Hessian), and only the vectors associated with the QPs vary across the sub-problems. We show that solving all the QP sub-problems in one shot reduces to computing one large matrix-vector product that can be trivially parallelized over GPUs using off-the-shelf linear algebra solvers.**Applied:** We release our entire implementation for review and to promote further research on this problem. We also release the benchmark data sets used in our simulations.**State-of-the-art Performance** We compare our GPU accelerated optimizer with two strong baselines (Park et al., 2020; Rastgar et al., 2021) and show massive improvement in computation time while being competitive in trajectory quality as measured by metrics like arc-length and smoothness. Our first comparison is with (Park et al., 2020) that uses a sequential approach for multi-robot trajectory optimization. Our computation time is at least 76.48% lower than that of (Park et al., 2020) for a smaller problem size involving 16 robots. Moreover, the performance gap increases substantially in our favor as we increase the number of robots and make the environment more cluttered by introducing more static obstacles. We observe similar trends in trajectory arc-length and smoothness comparison between the two approaches. Our second comparison is with (Rastgar et al., 2021) that searches directly in the feasible joint space formed by all the pair-wise collision avoidance constraints. Our proposed optimizer shows improved scalability over (Rastgar et al., 2021) for a larger number of robots while being also superior in trajectory arc length and smoothness.

## 2 Problem Formulation and Related Work

This section introduces the general problem formulation for multi-robot trajectory optimization. We subsequently use the problem set-up to review existing works and contrast our optimizer with them. We begin by summarizing the main symbols and notations used throughout the paper.

### 2.1 Symbols and Notations

In this paper, the lower normal and bold letters denote the scalars and vectors, respectively, while the upper bold case variants represent matrices. The left and right super-scripts denoted by *k* and *T* will be used to denote the iteration index of the optimizer and transpose of the vectors and matrices. The time-stamp of any variable will be denoted by *t*. The symbol ${\left\|.\right\|}_{2}$ stands for *l*_2_ norm. We summarize some of the main symbols in Table 1 while some are also introduced in their first place of use. At some places, we perform a special construction where time-stamped variables are stacked to form a vector. For example, **x**_*i*_ will be formed by stacking *x*_*i*_(*t*) at different time instants.

**TABLE 1:** Important symbols.

*n*_*p*_, *n*_*v*_, *n*_*r*_	Planning Steps, Number of Variables Parameterizing Trajectory along Each Motion Axis, and Number of Robots, Respectively
*a*, *b*	Spheroid dimensions
*x*_*i*_(*t*), *y*_*i*_(*t*), *z*_*i*_(*t*)	Position of *ith* robot at time *t*
${\bar{x}}_{i}\left(t\right),{\bar{y}}_{i}\left(t\right),{\bar{z}}_{i}\left(t\right)$	Predicted position of *jth* agent
***λ***_*i*_	Lagrangian multiplier

### 2.2 Robot Kinematics

Our optimizer is designed for robots with holonomic motion models. That is, the motion along each axis is decoupled from each other. This is a common assumption made in quadrotor motion planning. Many commercially available wheeled mobile robots also have similar kinematic model. Under certain conditions, even motion planning for car-like vehicles also adopt similar kinematic model and thus our optimizer is suitable for those as well Werling et al. (2010).

### 2.3 Trajectory Optimization

For holonomic robots modeled as series of integrators, the joint trajectory optimization can be formulated in the following manner.

$$\underset{x_{i}\left(t\right),y_{i}\left(t\right),z_{i}\left(t\right)}{\min}\underset{t,i}{\sum }\left({\ddot{x}}_{i}^{2}\left(t\right)+{\ddot{y}}_{i}^{2}\left(t\right)+{\ddot{z}}_{i}^{2}\left(t\right)\right),$$ (1a)

$$\left(x_{i}\left(t_{0}\right),{\dot{x}}_{i}\left(t_{0}\right),{\ddot{x}}_{i}\left(t_{0}\right),y_{i}\left(t_{0}\right),{\dot{y}}_{i}\left(t_{0}\right),{\ddot{y}}_{i}\left(t_{0}\right),z_{i}\left(t_{0}\right),{\dot{z}}_{i}\left(t_{0}\right),{\ddot{z}}_{i}\left(t_{0}\right)\right)=b_{o,i},\forall i$$ (1b)

$$\left(x_{i}\left(t_{f}\right),{\dot{x}}_{i}\left(t_{f}\right),{\ddot{x}}_{i}\left(t_{f}\right),y_{i}\left(t_{f}\right),{\dot{y}}_{i}\left(t_{f}\right),{\ddot{y}}_{i}\left(t_{f}\right),z_{i}\left(t_{f}\right),{\dot{z}}_{i}\left(t_{f}\right),{\ddot{z}}_{i}\left(t_{f}\right)\right)=b_{f,i},\forall i$$ (1c)

$$-{\left(\begin{array}{c} x_{i}\left(t\right)-x_{j}\left(t\right)\\ y_{i}\left(t\right)-y_{j}\left(t\right)\\ z_{i}\left(t\right)-z_{j}\left(t\right) \end{array}\right)}^{T}S\left(\begin{array}{c} x_{i}\left(t\right)-x_{j}\left(t\right)\\ y_{i}\left(t\right)-y_{j}\left(t\right)\\ z_{i}\left(t\right)-z_{j}\left(t\right) \end{array}\right)+1\leq 0,\forall t,\left\{i,j\in \left\{1,2,\ldots ,n_{r}\right\},j\neq i\right\},S=\left[\begin{array}{ccc} a^{2} & 0 & 0\\ 0 & a^{2} & 0\\ 0 & 0 & b^{2} \end{array}\right]$$ (1d)

The cost function Eq. 1a minimizes the squared norm of the acceleration at each time instant for all the robots. The equality constraints Eqs. 1b and 1c enforces the initial and final boundary conditions on positions, velocity, and accelerations on each robot trajectory. The pair-wise collision avoidance constraints are modeled by inequalities Eq. 1d, wherein we have assumed that the robots are shaped as axis-aligned spheroids with axis dimensions (*a*, *a*, *b*). For the ease of exposition, we consider all robots to have the same shape. Extension to a more general setting is trivial. The constraints Eq. 1d are typically enforced at pre-selected discrete time-stamps, and thus a fine resolution of discretization is necessary for accurately satisfying the constraints. For now, we do not consider any static obstacles in the environment in the formulation above. The extension is trivial as static obstacles can be considered robots with zero velocity and whose trajectories are not updated within the optimizer’s iteration.

Let the trajectory of each robot along each motion axis *x*, *y*, *z* be parameterized through *n*_*v*_ number of variables. For example, these variables could be time-stamped way-points representing the trajectory or the coefficients of their polynomial representation (see Eq. 8). Then, for a set-up with *n*_*r*_ number of robots and a planning horizon of *n*_*p*_, optimization Eqs. 1a–1d involves *n*_*r*_∗*n*_*v*_ variables, and 18∗*n*_*r*_ equality constraints. The number of pair-wise collision constraints would be nr2∗np .

The number of decision variables in optimization Eqs. 1a–1d scales linearly with the number of robots. Although this increase poses a computational challenge, the main difficulty in solving the optimization stems from the non-convex pair-wise collision avoidance constraints Eq. 1d as the rest of the cost and constraint functions are convex. Moreover, the number of collision avoidance constraints increases exponentially with the number of robots. Existing works (Augugliaro et al., 2012; Chen et al., 2015; Li et al., 2020; Park et al., 2020; Rastgar et al., 2021) have adopted different simplifications on the collision avoidance constraints to make multi-robot trajectory optimization more tractable. We thus next present a categorization of these works based on the exact methodology used.

### 2.4 Literature Review

#### 2.4.1 Joint Optimization With Conservative Convex Approximation

The most conceptually simple approach is to solve Eqs. 1a–1d as one large optimization problem, wherein the trajectory of every robot is computed in one shot. Authors in (Augugliaro et al., 2012) simplified the joint optimization by deriving a conservative affine approximation of the collision avoidance constraints Eq. 1d and consequently reducing Eqs. 1a–1d to a sequence of QPs. As a result, their solution process becomes somewhat tractable for a moderate number of robots (≈10) . However, the computation time of (Augugliaro et al., 2012) scales poorly because the number of affine constraints still increases exponentially with the number of robots. Our prior work (Rastgar et al., 2021) substantially improved the scalability of joint multi-robot trajectory optimization by reformulating the Euclidean collision constraints Eq. 1d into polar form and augmenting them into the cost function by using concepts from the Alternating Direction Method of Multipliers (ADMM). Moreover, we showed that such reduction allowed one-time offline caching of the most expensive parts of the computation. As a result (Rastgar et al., 2021), achieved over two orders of magnitude speed-up over (Augugliaro et al., 2012) for 16 robots. The current proposed work provides a further significant improvement over (Rastgar et al., 2021) in computation time and trajectory quality.

#### 2.4.2 Sequential Optimization

Sequential planners plan for only one robot at a time. At any given planning cycle, the previously computed robot trajectories are considered dynamic obstacles for the currently planned robot. As a result, these approaches ensure that the number of decision variables does not increase with robots. Moreover, the number of collision avoidance constraints increases linearly as the planning cycle progresses. However, note that the linear increase in the number of constraints does not translate to similar scaling in computation time. Even state-of-the-art interior-point solvers have cubic complexity with respect to the number of constraints.

A critical disadvantage of sequential planners is that each subsequent robot has access to less feasible space to maneuver. As a result, optimization problems become progressively constrained as the planning cycle progresses, leading to potential infeasibility. Authors in (Chen et al., 2015a) tackle this problem by developing an incremental constraint tightening approach. The authors integrate a subset of collision avoidance constraints into the optimization problem, and the size of this set is gradually increased based on the actual collision residuals.

Sequential planners naturally have the notion of priority, and these can be chosen carefully for improved performance. For example (Li et al., 2020), adopts a priority-based optimization method in which the robots are divided into groups/batches with pre-determined priorities, and the trajectory optimization problem is solved from the highest to the lowest priority group. Similar approach was adopted in (Park et al., 2020). Performing sequential planning over a small batch of robots reduces its conservativeness. On the other hand, it introduces an additional challenge of ensuring collision amongst the robots in a given batch. Authors in (Park et al., 2020) tackle this bottleneck by leveraging graph-based Multi-robot Path Finding (MAPF) methods.

#### 2.4.3 Distributed Optimization

Distributed optimizers at each iteration, break Eqs. 1a–1d into decoupled smaller problems. For example, see (Bento et al., 2013; Halsted et al., 2021). The key insight upon which all existing works build is that the only coupling between different robots stem from the pair-wise collision constraints Eq. 1d (Halsted et al., 2021). Thus, we if we discard this coupling, Eqs. 1a–1d can be easily reduced to *n*_*r*_ number of decoupled optimizations. One way to achieve the said decoupling is to let each robot make prediction of how the trajectories of other robots are going to look in the immediate next iterations and use that to simplify the collision avoidance constraints. More formally, let $\left({\bar{x}}_{j}\left(t\right),{\bar{y}}_{j}\left(t\right),{\bar{z}}_{j}\left(t\right)\right)$ be the predicted position of *jth* robot at time *t*. Then, the collision avoidance constraints can be simplified as Eq. 2.

$${\left(\begin{array}{c} x_{i}\left(t\right)-{\bar{x}}_{j}\left(t\right)\\ y_{i}\left(t\right)-{\bar{y}}_{j}\left(t\right)\\ z_{i}\left(t\right)-{\bar{z}}_{j}\left(t\right) \end{array}\right)}^{T}S\left(\begin{array}{c} x_{i}\left(t\right)-{\bar{x}}_{j}\left(t\right)\\ y_{i}\left(t\right)-{\bar{y}}_{j}\left(t\right)\\ z_{i}\left(t\right)-{\bar{z}}_{j}\left(t\right) \end{array}\right)+1\leq 0$$ (2)

**FIGURE 1:**
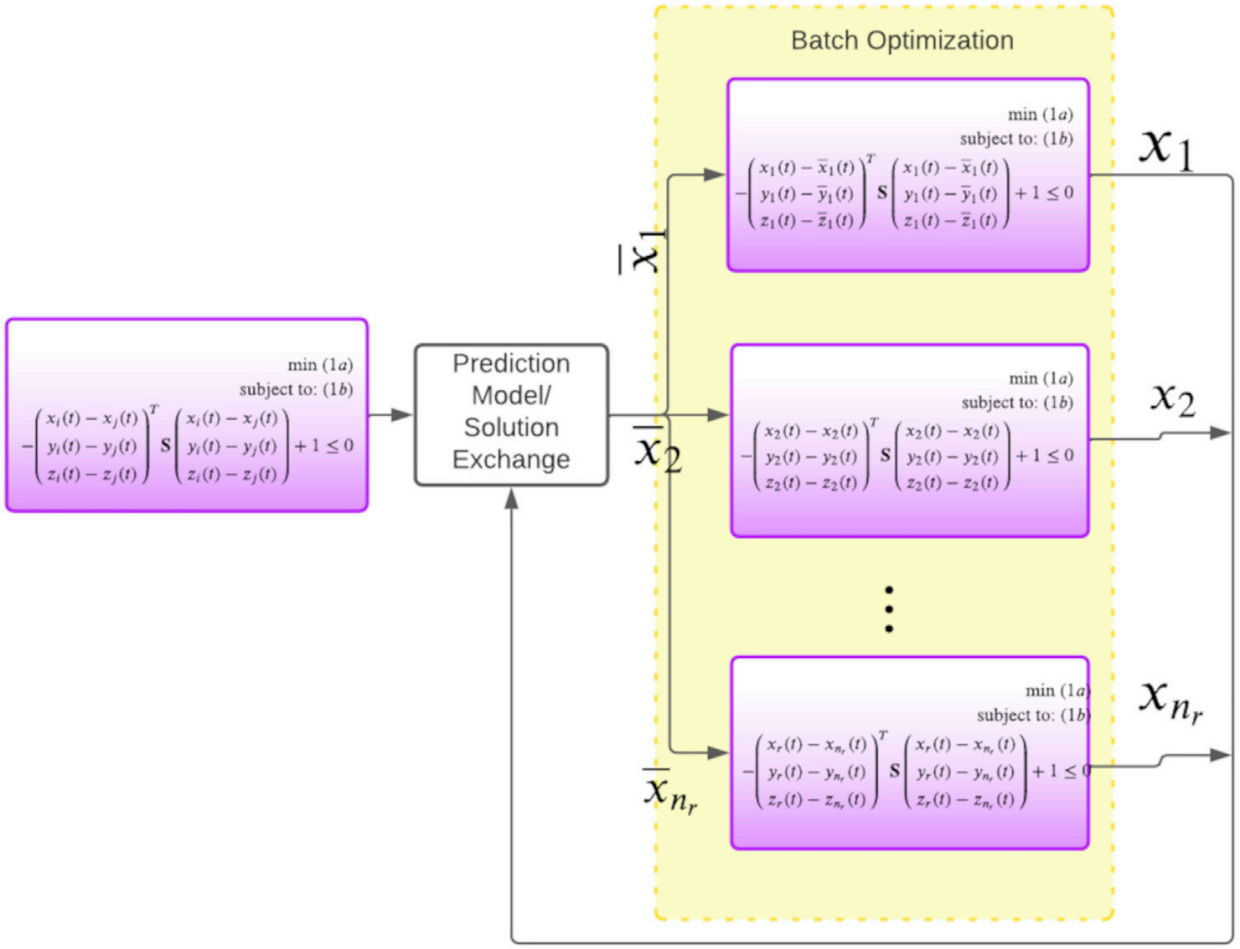
Figure shows our approach for breaking the joint optimization (first block) at each iteration into decoupled sub-problems (third group of blocks). Each robot exchanges their current computed trajectories with each other. For the next iteration, robots will use the communicated trajectories to frame their collision avoidance constraints. Thus, in this manner, we can avoid the inter-robot coupling in collision constraints. Our core novelty in this paper is a batch optimizer that can solve all the decoupled problems in parallel over GPUs. It is also possible to replace the trajectory exchange set-up with a model that predicts the nature of the robot trajectories in the immediate next iteration. Note that we only show the *x* component of the trajectory purely to maintain clarity in the figure.

Note that $\left({\bar{x}}_{j}\left(t\right),{\bar{y}}_{j}\left(t\right),{\bar{z}}_{j}\left(t\right)\right)$ is a known constant in Eq. 2. Figure 1 shows how the process of using Eq. 2 to formulate decoupled optimization problems for each robot. Existing works differ in their method of computing the prediction $\left({\bar{x}}_{j}\left(t\right),{\bar{y}}_{j}\left(t\right),{\bar{z}}_{j}\left(t\right)\right)$ . The simplest possibility is to set it as the solution obtained in the previous iteration (Bento et al., 2013), which is what we use in our formulation as well.

#### 2.4.4 Online Distributed Model Predictive Control

DMPC approaches are the online variants of the distributed optimization approach. In other words, if we run one iteration of distributed optimization and let each robot move with the computed trajectory, we recover the DMPC works such as (Luis et al., 2020), Luis and Schoellig (2019). This insight also points to the main issue of DMPC. At each control cycle, imagine a robot *i* receiving information (directly through communications or indirectly through prediction) about the trajectory that the other robot *j* computed. Then it uses this information to construct collision avoidance constraints in its trajectory optimization set-up. However, robot *j* will follow the same process and update its trajectory as well. Thus, essentially both robot *i* and *j* compute their motion based on outdated information about each other’s behavior.

#### 2.4.5 Batch Optimization Over CPU Vs. GPU

Parallelization of a batch of optimization problems across CPUs and GPUs operates fundamentally differently, and both classes of approaches have been tried in existing works to speed up multi-robot trajectory optimization. Each CPU core is efficient at handling arbitrary numerical computations, and thus solving a batch of optimizations problems in parallel is conceptually simple. We can solve each problem in a separate thread without needing to make any change in the underlying numerical algebra of the optimizer (Adajania et al., 2022), (Bento et al., 2013). As mentioned earlier, the scalability of CPU parallelization is limited by the number of cores (typical 6 in a standard laptop). On the other hand, GPUs have many cores, but these are primarily efficient at parallelizing primitive operations such as matrix-vector and matrix-matrix multiplication. Moreover, GPUs excel in performing the same primitive operations over many data points. Thus, to fully leverage the compute power of GPUs, it is necessary to modify the underlying numerical aspect of an optimizer to fit the strengths of GPUs. For example, GPU acceleration of Newton’s method requires adopting indirect matrix factorization over the more common direct approaches (Kylasa et al., 2019). One optimization technique that trivially accelerates over GPUs is Gradient Descent (GD) since it boils down to just matrix-vector multiplication. Authors in (Hamer et al., 2018) leverage this insight for developing a fast multi-robot trajectory optimization algorithm. One critical issue of (Hamer et al., 2018) is that the proposed GD is very sensitive to hyper-parameters like weights of the different cost function, learning rate, etc.

GPUs are designed using threads grouped into blocks, which are themselves organized as grids to parallelize computations for computational efficiency (Li et al., 2012). The GPU first tiles an *n* × *n* matrix using *p* × *q* tiles indexed with a 2-dimensional index to multiply large matrices. The output of each tile in the result matrix is independent of other tiles, which allows for parallelization. The parallelized CUDA code uses a block of threads to compute each tile of the result matrix, and to compute the entire result matrix; it uses a $\frac{n}{p}\times \frac{n}{q}$ grid of thread blocks. Many threads and blocks in modern GPUs allow for simultaneous computation of tile outputs, allowing for a many-fold boost in the computation time required for large matrix multiplications. Most off-the-shelf GPU-based libraries have this inbuilt CUDA programming for parallel GPU computations and can be utilized for achieving computational speed-ups in matrix multiplications.

## 3 Methods

### 3.1 Overview

Similar to (Bento et al., 2013), we break the joint multi-robot trajectory optimization Eqs. 1a–1d into decoupled smaller sub-problems at each iteration. This is illustrated in Figure 1. At a conceptual level, this decoupling process can be interpreted in the following manner: the robots communicate among themselves the trajectories they obtained in the previous iteration of the optimizer. Each robot then uses them to independently formulate their collision avoidance constraints. Our work differs from existing works in the way the decoupled problems illustrated in Figure 1 is solved. As mentioned before, a trivial approach to solving the sub-problems in parallel CPU threads is not scalable for tens of robots. In contrast, our main idea in this paper is to develop a GPU accelerated optimizer that can solve a batch of optimization problems in one shot.

In this sub-section, we aim to provide a succinct mathematical abstraction of our main idea. We discuss a special class of problems that are simple to solve in batch fashion. To this end, consider the following batch of equality constrained QPs, *i* ∈ {1, 2, …, *n*_*r*_}.

$$\underset{\xi_{i}}{\min}\left(\frac{1}{2}\xi_{i}^{T}\bar{Q}\xi_{i}+{\bar{q}}_{i}^{T}\xi_{i}\right),\;\text{st: }\bar{A}\xi_{i}={\bar{b}}_{i}$$ (3)

In total, there are *n*_*r*_ QPs to be solved, each defined over variable ***ξ***_*i*_. The QPs defined in Eq. 3 have a unique structure. The Hessian $\bar{Q}$ and the constrained matrix $\bar{A}$ are shared across the problems and only the vectors ${\bar{q}}_{i}$ and ${\bar{b}}_{i}$ varies across the batch. This special structure leads to efficient batch solution formulae. To see how, note that each QP in the batch can be reduced to solving the following set of linear equations.

$$\left[\begin{array}{cc} \bar{Q} & {\bar{A}}^{T}\\ \bar{A} & 0 \end{array}\right]\left[\begin{array}{c} \xi_{i}\\ \mu_{i} \end{array}\right]=\left[\begin{array}{c} {\bar{q}}_{i}\\ {\bar{b}}_{i} \end{array}\right],\;\forall i\in \left\{1,2,\ldots ,n_{r}\right\}$$ (4)

where ***μ***_*i*_ are the dual optimization variables. Now, it can be observed that the matrix on the left-hand side of Eq. 4 is independent of the batch index *i*, and thus, the solutions for the entire batch can be computed in one shot through Eq. 5.

$$\left[\begin{array}{c} \begin{array}{ccc} \xi_{1} & |\ldots | & \xi_{n_{r}}\\ \mu_{1} & |\ldots | & \mu_{n_{r}} \end{array} \end{array}\right]=\overset{\mathrm{matrix}}{\overset{︷}{\left({\left[\begin{array}{cc} \bar{Q} & {\bar{A}}^{T}\\ \bar{A} & 0 \end{array}\right]}^{-1}\right)}}\overset{\text{stacked vectors}}{\overset{︷}{\left[\begin{array}{c} \begin{array}{cccc} {\bar{q}}_{1} & {\bar{q}}_{2} & \ldots & {\bar{q}}_{n_{r}}\\ {\bar{b}}_{1} & {\bar{b}}_{2} & \ldots & {\bar{b}}_{n_{r}} \end{array} \end{array}\right]}},$$ (5)

where | represents that the columns are stacked horizontally. The batch solution Eq. 5 amounts to multiplying one single matrix with a batch of vectors. Furthermore, the matrix is constant, and its dimension is independent of the number of problems in the batch. Thus, operation Eq. 5 can be trivially parallelized over GPUs using off-the-shelf libraries like JAX (Bradbury et al., 2020).**How it all fits:** In the next few sub-sections, we will show how the distributed sub-problems of Eqs. 1a–1d, shown in Figure 1 can be solved efficiently in a batch setting. Specifically, we reformulate these problems in such a way that the most intensive part of their solution process reduces to solving a batch of QPs with the special structure presented in Eq. 3.

### 3.2 Collision Avoidance in Polar Form

An important building block of our approach is rephrasing the collision avoidance constraints into the following polar representation from (Rastgar et al., 2021; Rastgar et al., 2020).

$$f_{c}\left(x_{i}\left(t\right),y_{i}\left(t\right)\right)=\left\{\begin{array}{c} x_{i}\left(t\right)-{\bar{x}}_{j}\left(t\right)-ad_{ij}\left(t\right)\sin\beta_{ij}\left(t\right)\cos\alpha_{ij}\left(t\right)\\ y_{i}\left(t\right)-{\bar{y}}_{j}\left(t\right)-ad_{ij}\left(t\right)\sin\beta_{ij}\left(t\right)\sin\alpha_{ij}\left(t\right)\\ z_{i}\left(t\right)-{\bar{z}}_{j}\left(t\right)-bd_{ij}\left(t\right)\cos\beta_{ij}\left(t\right) \end{array}\right\},\;d_{ij}\left(t\right)\geq 1,$$ (6)

where *α*_*ij*_(*t*), *β*_*ij*_(*t*), *d*_*ij*_(*t*) are unknown variables that will be computed by the optimizer along with each robot’s trajectory. Physically, *α*_*ij*_(*t*) and *βij*(*t*) represent the 3D solid angle of the line-of-sight connecting robot *i* and *j* based on the predicted motion of the latter. The variable *d*_*ij*_(*t*) is the ratio of the length of the line-of-sight vector with minimum safe distance $\sqrt{a^{2}+a^{2}+b^{2}}$ (see (Rastgar et al., 2021)).

### 3.3 Proposed Reformulated Distributed Problem

Using Eq. 6, we can reformulate the distributed sub-problems presented in Figure 1 for the *ith* robot in the following manner. We reiterate that $\left({\bar{x}}_{j}\left(t\right),{\bar{y}}_{j}\left(t\right),{\bar{z}}_{j}\left(t\right)\right),\forall j\neq i$ is known based on the prediction of the trajectories of other robots.

$$\underset{x_{i}\left(t\right),y_{i}\left(t\right),z_{i}\left(t\right),d_{ij}\left(t\right),\alpha_{ij}\left(t\right),\beta_{ij}\left(t\right)}{\min}\underset{t}{\sum }\left({\ddot{x}}_{i}^{2}\left(t\right)+{\ddot{y}}_{i}^{2}\left(t\right)+{\ddot{z}}_{i}^{2}\left(t\right)\right),$$ (7a)

$$\left(x_{i}\left(t_{0}\right),{\dot{x}}_{i}\left(t_{0}\right),{\ddot{x}}_{i}\left(t_{0}\right),y_{i}\left(t_{0}\right),{\dot{y}}_{i}\left(t_{0}\right),{\ddot{y}}_{i}\left(t_{0}\right),z_{i}\left(t_{0}\right),{\dot{z}}_{i}\left(t_{0}\right),{\ddot{z}}_{i}\left(t_{0}\right)\right)=b_{o,i},\forall i$$ (7b)

$$\left(x_{i}\left(t_{f}\right),{\dot{x}}_{i}\left(t_{f}\right),{\ddot{x}}_{i}\left(t_{f}\right),y_{i}\left(t_{f}\right),{\dot{y}}_{i}\left(t_{f}\right),{\ddot{y}}_{i}\left(t_{f}\right),z_{i}\left(t_{f}\right),{\dot{z}}_{i}\left(t_{f}\right),{\ddot{z}}_{i}\left(t_{f}\right)\right)=b_{f,i},\forall i$$ (7c)

$$f_{c}\;:\;\left\{\begin{array}{c} x_{i}\left(t\right)-{\bar{x}}_{j}\left(t\right)-ad_{ij}\left(t\right)\sin\beta_{ij}\left(t\right)\cos\alpha_{ij}\left(t\right)\\ y_{i}\left(t\right)-{\bar{y}}_{j}\left(t\right)-ad_{ij}\left(t\right)\sin\beta_{ij}\left(t\right)\sin\alpha_{ij}\left(t\right)\\ z_{i}\left(t\right)-{\bar{z}}_{j}\left(t\right)-bd_{ij}\left(t\right)\cos\beta_{ij}\left(t\right) \end{array}\right\}$$ (7d)

$$d_{ij}\left(t\right)\geq 1,\;\forall t,j,\left\{j|j\in \left\{1,2,\ldots ,n_{r}\right\},j\neq i\right\}$$ (7e)

### 3.3.1 Finite Dimensional Representation

Optimization Eqs. 7a–7e is expressed in terms of functions and thus has the so called infinite dimensional representaton. To obtain a finite-dimensional form, we assume some parametric form for this functions. For different $d_{ij}\left(t\right),\alpha_{a_{ij}}\left(t\right),\beta_{ij}\left(t\right)$ , we assume a way-point paramterization. That is, these functions are represented through values at discrete time instants. The trajectories along each motion axis are represented as following polynomials.

$$\left[\begin{array}{c} x_{i}\left(t_{1}\right)\\ x_{i}\left(t_{2}\right)\\ \vdots \\ x_{i}\left(t_{n_{p}}\right) \end{array}\right]=Pc_{x,i},\;\left[\begin{array}{c} {\dot{x}}_{i}\left(t_{1}\right)\\ {\dot{x}}_{i}\left(t_{2}\right)\\ \vdots \\ {\dot{x}}_{i}\left(t_{n_{p}}\right) \end{array}\right]=\dot{P}c_{x,i},\;\left[\begin{array}{c} {\ddot{x}}_{i}\left(t_{1}\right)\\ {\ddot{x}}_{i}\left(t_{2}\right)\\ \vdots \\ {\ddot{x}}_{i}\left(t_{n_{p}}\right) \end{array}\right]=\ddot{P}c_{x,i}.$$ (8)

Similar expressions as Eq. 8 can be written for the *y*, *z* component of the trajectory as well. The matrix **P** is formed with time dependent polynomial basis functions. Using Eq. 8, we can re-write Eqs. 7a–7e in the following matrix form.

$$\underset{\xi_{1,i},\xi_{2,i},\xi_{3,i},\xi_{4,i}}{\min}\left(\frac{1}{2}\xi_{1,i}^{T}Q\xi_{1,i}\right),$$ (9a)

$$A_{eq}\xi_{1,i}=b_{eq},$$ (9b)

$$F\xi_{1,i}=g_{i}\left(\xi_{2,i},\xi_{3,i},\xi_{4,i}\right),$$ (9c)

$$\xi_{4,i}\geq 1,$$ (9d)

where, ***ξ***_1,*i*_ = (**c**_*x*,*i*_, **c**_*y*,*i*_, **c**_*z*,*i*_), ***ξ***_2,*i*_ = ***α***_*ij*_, ***ξ***_3,*i*_ = ***β***_*ij*_ and ***ξ***_4,*i*_ = **d**_*ij*_. Note that ***α***_*ij*_ is formed by stacking *α*_*ij*_(*t*) at different time instants. Similar construction is followed for other elements in ***ξ***_2_, ***ξ***_3_. The matrix **Q** is block diagonal matrix with ${\ddot{P}}^{T}\ddot{P}$ as main diagonal block. The affine constraint Eq. 9b is a matrix representation of the initial and final boundary conditions Eqs. 7b and 7c. The matrix **A**_*eq*_ and vector **b**_*eq*_ is constructed in the following manner.

$$A_{eq}=\left[\begin{array}{cc} A & 0\\ 0 & A \end{array}\right],A_{eq}={\left[\begin{array}{c} P_{1}|{\dot{P}}_{1}|{\ddot{P}}_{1}|P_{-1}|{\ddot{P}}_{-1}{\ddot{P}}_{-1} \end{array}\right]}^{T},b_{eq}=\left[\begin{array}{c} b_{0,i}\\ b_{f,i}, \end{array}\right]$$ (10)

where, $P_{1},{\dot{P}}_{1},{\ddot{P}}_{1},P_{-1},{\dot{P}}_{-1},{\ddot{P}}_{-1}$ represents the first and last elements of the corresponding matrices.

Matrix **F** and vector **g**_*i*_ defining constraints Eq. 7e are constructed as

$$F=\left[\begin{array}{ccc} F_{o} & 0 & 0\\ 0 & F_{o} & 0\\ 0 & 0 & F_{o} \end{array}\right]\;g_{i}=\left[\begin{array}{c} g_{x,i}\left(\xi_{2,i},\xi_{3,i},\xi_{4,i}\right)\\ g_{y,i}\left(\xi_{2,i},\xi_{3,i},\xi_{4,i}\right)\\ g_{z,i}\left(\xi_{2,i},\xi_{3,i},\xi_{4,i}\right) \end{array}\right]$$ (11)

where,

$$g_{x,i}={\bar{x}}_{j}+ad_{ij}\sin\beta_{ij}\cos\alpha_{ij},\forall j,\;g_{y,i}={\bar{y}}_{j}+ad_{ij}\sin\beta_{ij}\sin\alpha_{ij},\forall j,\;g_{z,i}={\bar{z}}_{j}+bd_{ij}\cos\beta_{ij},\forall j$$ (12)

and **F**_*o*_ is formed by vertically stacking **P**, *n*_*r*_ − 1 times. The vectors ${\bar{x}}_{j},{\bar{y}}_{j},{\bar{z}}_{j}$ are formed by stacking ${\bar{x}}_{j}\left(t\right),{\bar{y}}_{j}\left(t\right),{\bar{z}}_{j}\left(t\right)$ at different time instants.

**REMARK 1**. The subscript *i* signifies that Eqs. 9a–9c is constructed for the *ith* agent.

**REMARK 2**. All the non-convexity in optimization Eqs. 9a–9d is rolled into the equality constraint Eq. 9c.

**REMARK 3**. The matrices **A**_*eq*_, **F** in optimization Eqs. 9a–9d is independent of the robot index. In other words, these matrices remain the same irrespective of the which sub-problems shown in Figure 1 we are solving.

Remark 3 sheds light behind our motivation of presenting the elaborate reformulations of the collision avoidance constraints. In fact, on the surface, our chosen representation Eq. 6 seems substantially more complicated than the conventional form Eq. 1d based on the Euclidean norm. In the next sub-section, we present an optimizer that can leverage the insights presented in Remark 3. More precisely, we will show that the due to the matrices **A**_*eq*_, **F** being independent of the robot index *i*, the most intensive part of solving Eqs. 9a–9d reduces to the batch QP structure presented in Section 3.1.

### 3.4 Augmented Lagrangian and Alternating Minimization

Our proposed optimizer for Eqs. 9a–9d relies on relaxing the non-convex equality constraints Eq. 9c as *l*_2_ penalties and incorporating them into the cost function in the following manner.

$$\underset{\xi_{1,i},\xi_{2,i},\xi_{3,i},\xi_{4,i}}{\min}\left(\frac{1}{2}\xi_{1,i}^{T}Q\xi_{1,i}-\left\langle \lambda_{i},\xi_{1,i}\right\rangle +\frac{\rho }{2}{\left\|F\xi_{1,i}-g_{i}\left(\xi_{2,i},\xi_{3,i},\xi_{4,i}\right)\right\|}_{2}^{2}\right)$$ (13)

As the residual of the constraint term is driven to zero, we recover the solution to the original problem. To this end, the parameter ***λ***_*i*_, known as the Lagrange multiplier, plays an important part. Its role is to appropriately weaken the effect of the primary cost function so that the optimizer can focus on minimizing the constraint residual (Taylor et al., 2016). The parameter *ρ* is a scalar and is typically constant. However, it is possible to increase or decrease it depending on the magnitude of the constraint residual at each iteration of the optimizer.

The relaxation of non-convex equality constraints, as augmented Lagrangian (AL) cost, is extensively used in non-convex optimization (Ferranti and Keviczky, 2017; Ferranti et al., 2018). However, what differentiates our use of AL from existing works is how we minimize Eq. 13. Typical approaches towards non-convex optimization are based on first (and sometimes second) order Taylor Series expansion of the non-convex costs or constraints. In contrast, we adopt an Alternating Minimization (AM) based approach, wherein at each iteration, we minimize only one of the variable blocks amongst ***ξ***_1,*i*_, ***ξ***_2,*i*_, ***ξ***_3,*i*_, ***ξ***_4,*i*_ while others are held constant at specific values. In the next section, we present the various steps of our AM optimizer and highlight how it never requires any linearization of cost or constraints. Moreover, we show how the AM steps naturally lead to a simple yet efficient batch update rule using which we can solve Eqs. 9a–9d for all the robots in one shot.

Algorithm 1 Alternating Minimization Based Solution for the *Ith* Sub-Problem

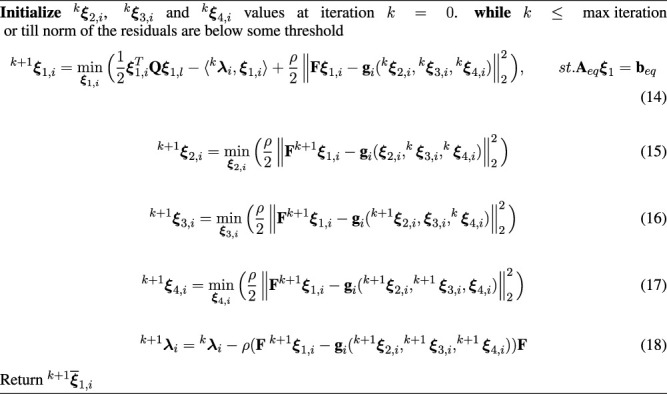

### 3.5 AM Steps and Batch Update Rule

Our AM based optimizer for minimizing Eq. 13 subject to Eqs. 9b–9d is presented in Algorithm 1. Here, the left superscript *k* is used to track the values of the variable across iteration. For example, ^*k*^ ***ξ***_2,*i*_ denotes the value of this respective variable at iteration *k*.

The Algorithm begins (line 1) by providing the initial guesses for ***ξ***_2,*i*_, ***ξ***_3,*i*_, ***ξ***_4,*i*_. The main optimizer iterations run within the while loop for the specified max iteration limit or till the constraint residuals are a below specified threshold. Each step within the while loop involves solving a convex optimization over just one variable block. We present a more detailed analysis of each of the steps next.

#### 3.5.1 Analysis

**Step (14):** This optimization is a convex QP with a similar structure as Eq. 3 with

Q¯=Q+ρFTF,q¯i=−λik−ρFTgiξ2,ik,ξ3,ik,ξ4,ikT. (19)

Thus, we can easily solve Eq. 14 for all the robots in parallel to obtain $\left(\xi_{1,1},\xi_{1,2},\xi_{1,3},\ldots ,\xi_{1,n_{r}}\right)$ in one shot. The exact solution update is given by Eq. 5.

For a constant *ρ*, the inverse of $\bar{Q}$ needs to be obtained only once irrespective of the number of robots. Thus, the complexity of the batch solution of all the sub-problems stems purely from obtaining the matrix-matrix products in Eq. 5 and **F** ^*T*^ **g**_*i*_, *∀i*. We can formulate the latter also as one large matrix-matrix product in the following manner.

$$F^{T}(\overset{G}{\overset{︷}{\left([g_{1}|g_{2}|\ldots |g_{n_{r}}\right]})}{\left.\right)}^{T}$$ (20)

The dimension of **F**, **g**_*i*_ and **G** is ((*n*_*r*_ − 1)∗*n*_*p*_) × 3*n*_*v*_, ((*n*_*r*_ − 1)*n_*p*_) × 1, and ((*n*_*r*_ − 1)*n_*p*_) × *n*_*r*_ respectively. For convenience, we recall that *n*_*r*_, *n*_*p*_, *n*_*v*_ represents the number of robots, planning steps and coefficients of the trajectory polynomial (along each axis) respectively. Thus, the row-dimension of **F** and **G** increases linearly with *n*_*r*_.

**Step (15):** The variable ^*k*+1^ ***ξ***_1,*i*_ computed in the previous step and Eq. 8 can be used to fix the position trajectory ^*k*+1^ ***x***_*i*_, ^*k*+1^ ***y***_*i*_, ^*k*+1^ ***z***_*i*_ at the (*k* + 1)^*th*^ iteration. Thus, optimization Eq. 15 reduces the following form

∀i,j,αijk+1=minαijρ2xik+1−x¯j︷x~ik+1−adijksinβij⁡cosαijyik+1−y¯j︷y~ik+1−adijksinβij⁡sinαij22 (21)

where ${\bar{x}}_{j},{\bar{y}}_{j}$ is formed by stacking ${\bar{x}}_{j}\left(t\right),{\bar{y}}_{j}\left(t\right)$ at different time instants.

Although Eq. 21 is a seemingly non-convex problem but it has a few favorable computational structures. First, for a fixed position trajectory ^*k*+1^ ***x***_*i*_, ^*k*+1^ ***y***_*i*_, ^*k*+1^ ***z***_*i*_, we can treat each element of ***α***_*ij*_ as independent from each other. Thus, Eq. 21 reduces to (*n*_*r*_ − 1)*n_*p*_ decoupled problems. Second, the solution can be obtained by purely geometrical intuition; ***α***_*ij*_ is simply one part of the 3D solid-angle of the line-of-sight connecting the *ith* robot and the predicted trajectory of *jth* agent. The exact solution update is given by the following.

ξ2,ik+1=αijk+1=arctan⁡2y~ik+1,x~ik+1, (22)

**Step (16):** Following the exact same reasoning as the previous step (15), we have the following solution update rule for ***ξ***_3,*i*_:

ξ3,i=βijk+1=arctan⁡2x~ik+1,a⁡cosαijk+1,z~ik+1b (23)

**Step** ^***k*+1**^ ***ξ***_**4,*i***_ **:** Similar to the last two steps, each element of ***ξ***_4,*i*_ = **d**_*ij*_ once the position trajectory ^*k*+1^ ***x***_*i*_, ^*k*+1^ ***y***_*i*_, ^*k*+1^ ***z***_*i*_ is fixed. Thus, Eq. 17 can be broken down into (*n*_*r*_ − 1)*n_*p*_ parallel problems of the following form.

ξ4,ik+1=dijk+1=mindij≥1ρ2xik+1−xj︷x~ik+1−adij⁡sinβijk+1cosk+1αijyik+1−yj︷y~ik+1−adij⁡sinβijk+1sinαijk+1zik+1−zj︷z~ik+1−bdij⁡cosβijk+122 (24)

Each optimization in Eq. 24 is a single variable QP with simple bound constraints. We first obtain the symbolic formulae for the unconstrained version and then clip the resulting solution to [0 1].

**REMARK 4.** Evaluating Eqs. 22 and 23 and the solution of Eq. 24 requires no matrix factorization/inverse or even matrix-matrix products. We just need element-wise operation that can obtained for all the sub-problems in one shot. In other words, we obtain $\left(\xi_{2,1},\xi_{2,2},\ldots \xi_{2,n_{r}}\right)$ , $\left(\xi_{3,1},\xi_{3,2},\ldots \xi_{3,n_{r}}\right)$ , and $\left(\xi_{4,1},\xi_{4,2},\ldots \xi_{4,n_{r}}\right)$ in parallel.

## 4 Results

The objective of this section is twofold. First, to validate that a distributed approach augmented with our custom batch optimizer can indeed generate collision-free trajectories for tens of robots in highly cluttered environments. Second, to compare our approach with the existing state-of-the-art (SOTA) multi-robot trajectory optimizer in terms of solutions quality and computation time.

**Implementation Details:** We built our optimizer in *Python* using JAX (Bradbury et al., 2020) as our GPU accelerated linear algebra back-end. We considered static obstacles as robots with fixed zero velocity. We modeled each robot by a sphere and each obstacle by its circumscribing sphere. We experiment with a diverse range of radii of both robots and obstacles. Simulations were run on a desktop computer with 32 GB RAM and RTX 2080 NVIDIA GPU.

### 4.1 Benchmarks and Convergence

Our optimizer is tested using the following benchmarks.

• The robots’ start and goal positions are sampled along the circumference of a circle.
• The robots are initially located on a grid and are tasked to converge to a line formation.

**FIGURE 2:**
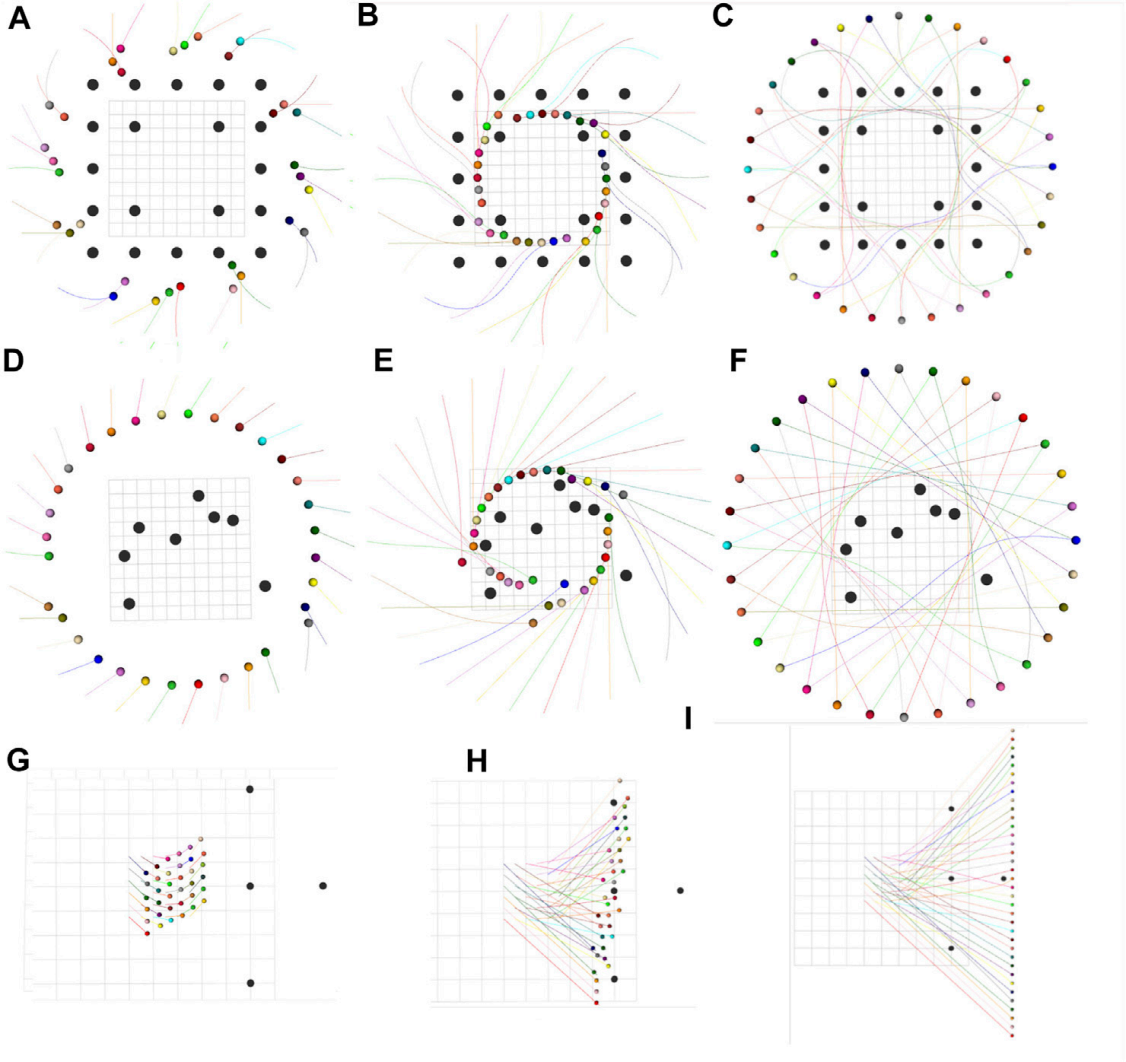
Trajectory snapshots for **(A–C)** 32 robots, each of radius 0.3 m arranged in a circle and 20 obstacles of radius 0.4 m **(D–F)** 32 robots, each of radius 0.3 m arranged in a circle and 8 randomly placed obstacles of radius 0.4 m **(G–I)** 36 robots, each of radius 0.1 m arranged in a grid configuration are required to move to a line formation. Also, the environment has 4 static obstacles, each of radius 0.15 m.

**FIGURE 3:**
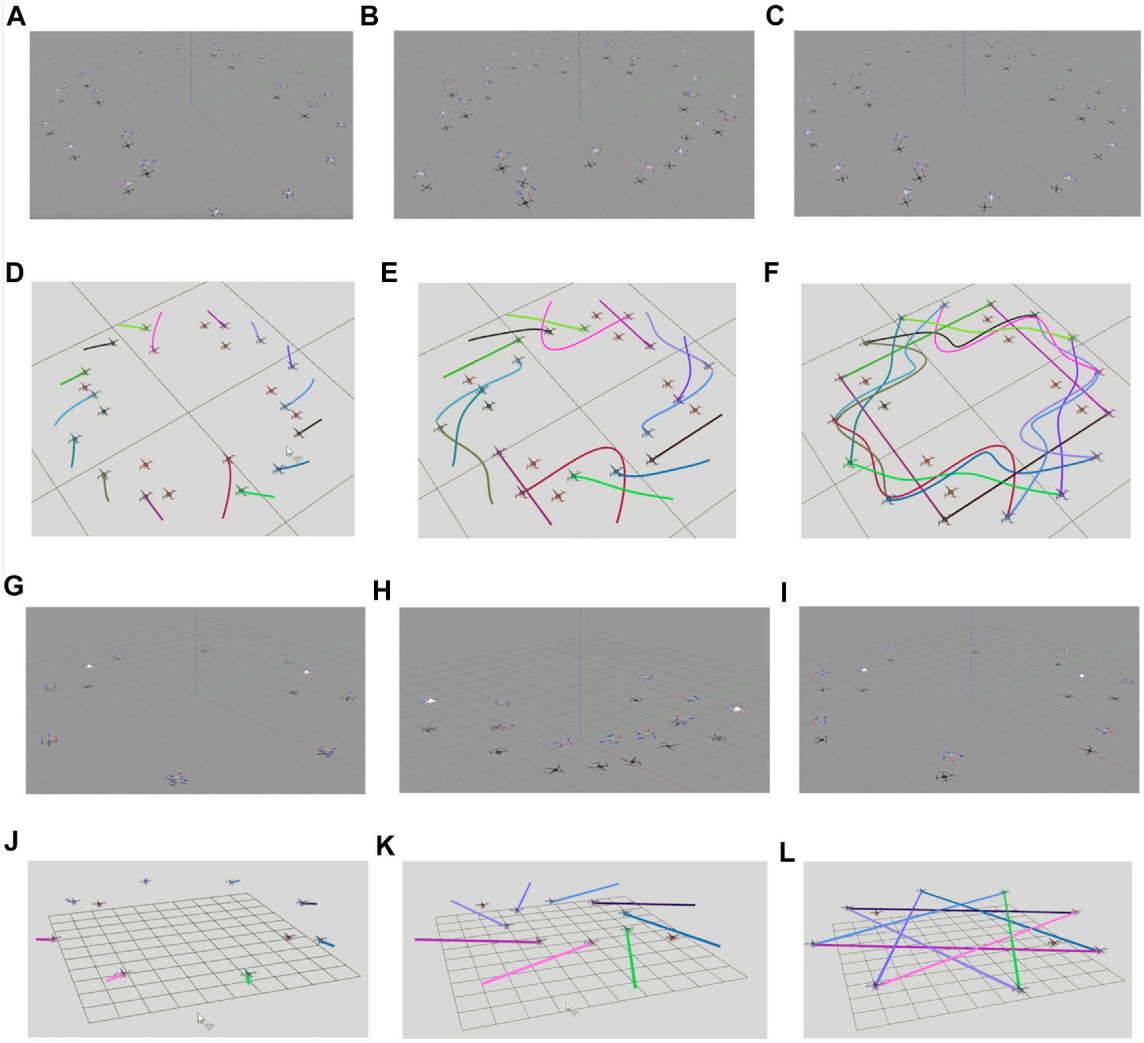
Simulation snapshots for **(A–F)** 16 drones and 8 static obstacles of radius 0.3 m, and **(G–L)** 8 drones and 2 static obstacles of radius 0.3 m **(A–C, G–I)** are screenshots of simulations on Gazebo. In **(A–C)**, the gray static hovering drones represent static obstacles while in **(G–I)** white hovering drones represent static obstacles. **(D–F, J–L)** are screenshots of RViz simulations with the brown hovering drones representing static obstacles. For the full simulation videos, please refer to the following link: https://www.dropbox.com/scl/fo/xnostapkvf72uudyb840t/h?dl=0&rlkey=02gjjllohbomzi0kcifbqezt9.

By changing the number and positions of robots and static obstacles, we created several variations of the mentioned benchmarks and utilized them to validate our optimizer. Figures 2A–I presents a few qualitative results in a diverse set of environments. Figures 2A–C shows an environment with 32 robots and 20 obstacles. Interestingly, we observe a circular pattern formation among the robots while passing through narrow passages between static obstacles. In Figures 2D–F, 36 robots initially arranged in a grid are given the task to navigate to a line formation while avoiding collisions with each other and with the four static obstacles in the environment. Figure 3 shows the execution of the computed trajectories in a high-fidelity physics engine called Gazebo available in Robot Operation System (Koenig and Howard, 2004).

**FIGURE 4:**
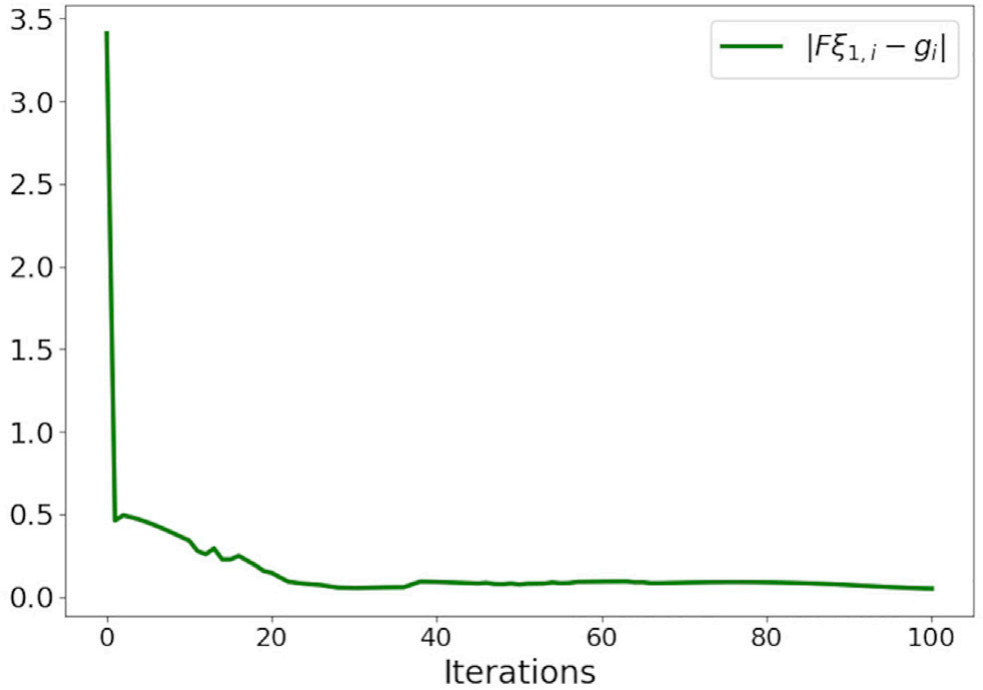
Empirical validation of convergence of our optimizer. The figure shows the residual of ‖**F*ξ***_1,*i*_ − **g**_*i*_‖ averaged over all *i* (agent index) and across different benchmarks.

A conceptually simple way of validating the convergence of the proposed optimizer is to observe the trends in residual of constraints Eq. 9c over iterations. If the residuals converge to zero, the computed trajectories are guaranteed to be collision-free. Figure 4 empirically provides this validation. It presents ‖**F*ξ***_1,*i*_ − **g**_*i*_‖ averaged over all *i*. Furthermore, we average the residuals over different trials in various benchmarks. We can observe from Figure 4 that, on average, 100 iterations are sufficient to obtain residuals around 0.01. A further increase in residuals can be obtained by increasing the number of iterations but at the expense of increasing the computation time. In our implementation, we adopt a heuristic wherein we inflate the size of the robots with four times the typical residual observed after 100 iterations.

**FIGURE 5:**
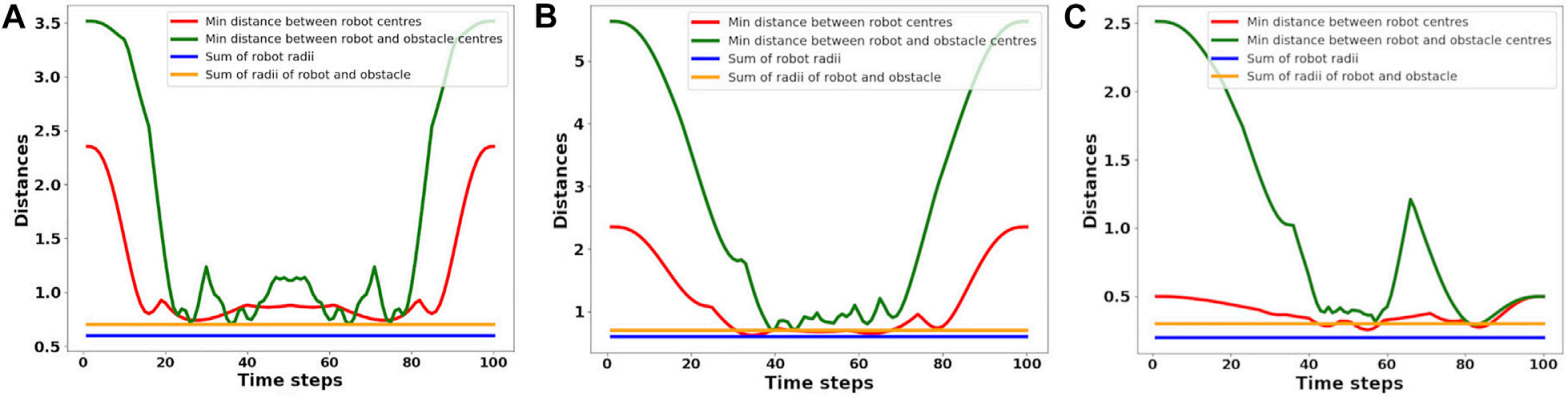
Figure showing the minimum of the pair-wise distances between the robots averaged across different benchmarks, some of which are shown in Figure 2. The pair-wise distance is always greater than the lower bound shown in blue. Similarly, we also show the average minimum distance between the robots’ and obstacles’ centers. The corresponding lower bound is shown in yellow which is always respected by the computed trajectories. **(A)** denotes the pairwise-distance plot for the scenario in Figures 2A–C, **(B)** for Figures 2D–F and **(C)** for Figures 2G–I.

For a further sanity check, we check for inter-robot and robot-obstacle distances at each point along the computed trajectories (Figure 5). Collisions are considered to have happened if the distances are less than sum of the robots’ (blue line in Figure 5) or robot-obstacles’ (yellow line in Figure 5) radii. Figure 5 summarizes the average behavior observed across several trials, which validates the satisfaction of the collision avoidance requirement.

### 4.2 Comparisons With State-Of-The-Art

This subsection compares our optimizer with existing state-of-the-art approaches (Rastgar et al., 2021) and (Park et al., 2020). We use the following metrics for bench-marking.

• Smoothness cost: It is computed as the norm of the second-order finite-difference of the robot position at different time instances.
• Arc-length: It is computed as the norm of the first-order finite-difference of the robot positions at different time instances.
• Computation Time: The time taken for each approach to return a smooth and collision-free solution.

#### 4.2.1 Comparison With (Park et al., 2020)

**FIGURE 6:**
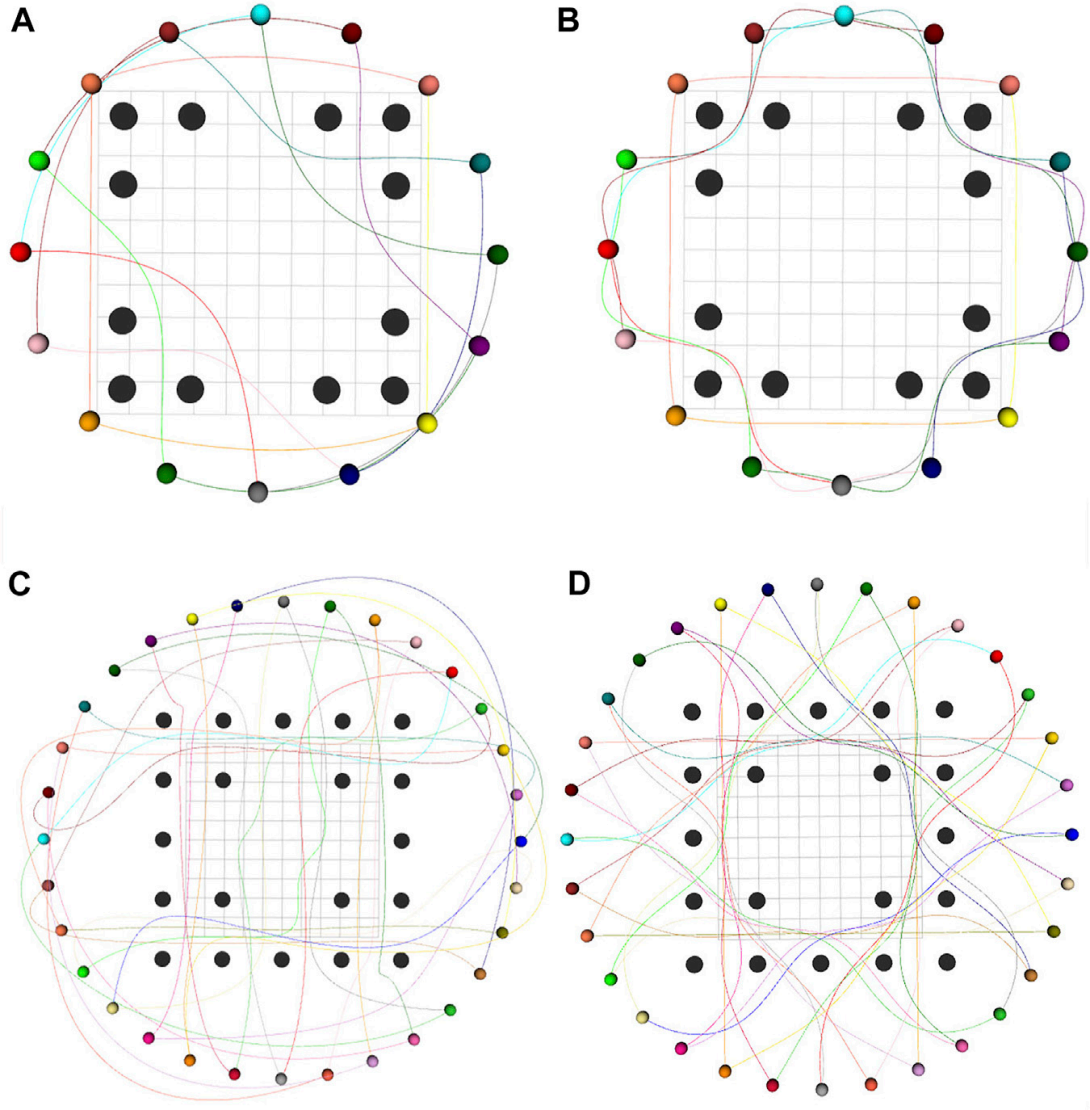
Comparison of trajectories generated by (Park et al., 2020) **(A,C)** and our optimizer **(B,D)** for 16 robots-12 obstacles (upper row) and 32 robots-20 obstacles (bottom row) benchmarks. Black spheres denote static obstacles and colored spheres denote robots. Our optimizer generates trajectories with smaller arc-length than (Park et al., 2020).

**FIGURE 7:**
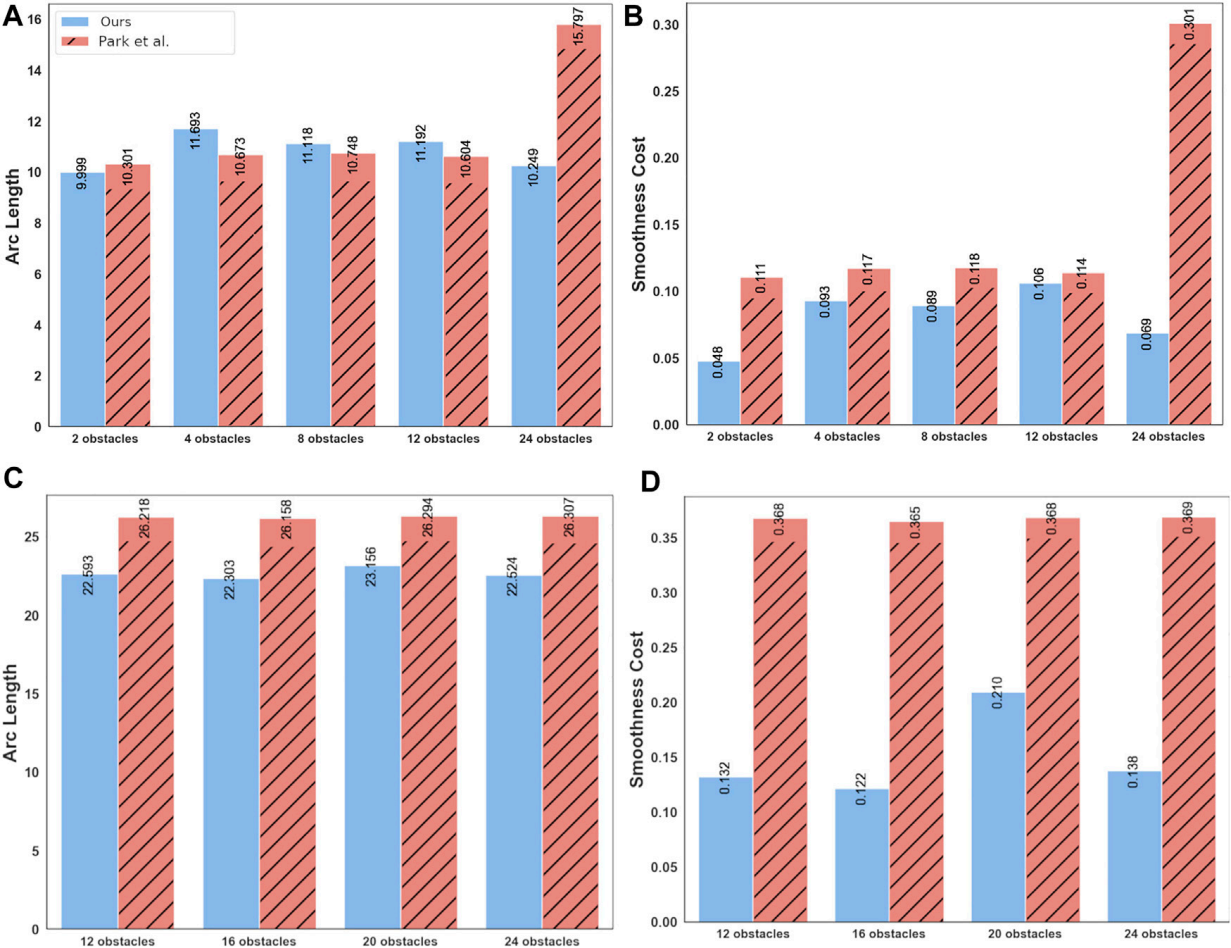
Comparison of our optimizer with (Park et al., 2020) in terms of arc-length and smoothness of obtained trajectories in 16 robots **(A,B)** and 32 robots **(C,D)** benchmarks. Our optimizer generates trajectories with not only better smoothness, but also with shorter arc-lengths. Moreover, the performance gap between our approach and (Park et al., 2020) increases as the environment becomes more cluttered.

Figure 6 presents a qualitative comparison between the trajectories obtained by our optimizer and (Park et al., 2020) in two different benchmarks. Both approaches are successful; however, ours results in shorter trajectories. This trend is further confirmed by Figure 7. Our optimizer achieves an average reduction of 3.90 and 13.72% in arc-length in 16 and 32 robots benchmarks, respectively. Furthermore, the performance gap between our optimizer and (Park et al., 2020) increases as the environment becomes more cluttered with static obstacles. We also observe similar trends in the smoothness metric, with the performance gap being even starker. Our optimizer achieves an average reduction of 35.86 and 59.06% in smoothness cost in 16 and 32 robots benchmark, respectively.

Table 2 compares the computation time of our optimizer and (Park et al., 2020). Our optimizer shows better scaling with the number of robots and obstacles in the environment. On the considered benchmark, our optimizer shows a worst and best case improvement of 74.28 and 98.48% respectively. The trends in computation time can be understood in the following manner. The approach of (Park et al., 2020) uses sampling-based multi-agent pathfinding algorithms to compute initial guesses for the robot trajectories. As the environment becomes more cluttered, the computational cost of computing the initial trajectories increases dramatically. Moreover, their sequential optimization also becomes increasingly more computationally intensive as the number of robots and obstacle increase.

**TABLE 2:** Comparison with current state-of-the-art (Park et al., 2020) in terms of computation time.

Number of Robots	Number of obstacles	ours [s]	(Park et al., 2020)[s]
32	24	0.21	12.897
32	20	0.20	11.827
32	16	0.20	12.423
32	12	0.19	12.504
16	24	0.17	0.795
16	12	0.17	0.661
16	8	0.16	0.680
16	4	0.16	0.702
16	2	0.15	0.621

In contrast, our optimizer only requires matrix-matrix products, and the dimension of these matrices increases linearly with the number of robots and obstacles. This linear scaling along with GPU parallelization explains our computation time.

#### 4.2.2 Comparison With (Rastgar et al., 2021)

Table 3 compares the performance of our optimizer with (Rastgar et al., 2021). Our core difference with (Rastgar et al., 2021) stems from the fact that we break a large optimization problem into smaller distributed sub-problems. In contrast, (Rastgar et al., 2021), retains the original larger problem itself. However, both our optimizer and (Rastgar et al., 2021) use GPUs to accelerate the underlying numerical computations. Thus, unsurprisingly, (Rastgar et al., 2021), shows a decent scaling with the number of robots and obstacles. Nevertheless, our approach still outperforms (Rastgar et al., 2021). Specifically, in 16 robot benchmarks, our optimizer shows a worst-case improvement of 2 times over (Rastgar et al., 2021) in computation time. As the environment becomes more cluttered, this factor increases to almost 10. In 32 robot benchmarks, the difference between our optimizer and (Rastgar et al., 2021)’s computation time is around nine times.

**TABLE 3:** Comparison with (Rastgar et al., 2021) in terms of computation time, arc-length and smoothness cost.

Number of Robots	Number of obstacles	Benchmark	Computation time [s]	Arc-length [M]	Smoothness Cost
16 robot	2	Rastgar et al. (2021)	0.34	13.488	0.102
	2	Ours	0.15	9.999	0.048
	4	Rastgar et al. (2021)	0.37	14.257	0.114
	4	Ours	0.16	11.693	0.093
	8	Rastgar et al. (2021)	0.70	15.539	0.140
	8	Ours	0.16	11.118	0.089
	12	Rastgar et al. (2021)	0.79	15.931	0.159
	12	Ours	0.17	11.192	0.106
	24	Rastgar et al. (2021)	1.49	24.198	0.164
32 robots	24	Ours	0.17	10.249	0.069
	12	Rastgar et al. (2021)	1.688	23.855	0.157
	12	Ours	0.19	22.593	0.132
	16	Rastgar et al. (2021)	1.752	24.04	0.164
	16	Ours	0.20	22.303	0.122
	20	Rastgar et al. (2021)	1.804	24.14	0.170
	20	Ours	0.20	23.156	0.210

In terms of the arc-length and the smoothness metrics, our optimizer shows an improvement of around 57 and 58% respectively over (Rastgar et al., 2021). However, both approaches provide comparable results in the more challenging 32 robot benchmarks. The arc-length and smoothness cost difference decreases the environment becomes more cluttered.

## 5 Discussions and Future Work

Joint multi-robot trajectory optimizations are generally considered intractable beyond a small number of robots. This is because the number of pair-wise collision avoidance constraints increases exponentially with the number of robots. Moreover, even the best optimization (QP) solvers show polynomial scaling with the number of constraints. In this paper, we fundamentally altered this notion. By employing a clever set of reformulations and parallelism offered by modern computing devices such as GPUs, we managed to compute trajectories for tens of robots in highly cluttered environments in a fraction of a second. Our formulation is simple to implement and involves computing just matrix-matrix products. Such computations can be trivially accelerated or parallelized on GPUs using off-the-shelf libraries like JAX (Bradbury et al. (2020)). We benchmarked our approach against two strong baselines and showed substantial improvement over them in terms of computation time and trajectory quality.

Our work has potential beyond multi-robot coordination. For example, currently, we are looking to use the proposed multi-robot trajectory optimization for interaction-aware trajectory prediction. Our optimizer’s current form is suited for only holonomic robots. In future works, we are looking to integrate non-holonomic constraints to make our optimizer applicable for coordination of car-like vehicles.

## Data Availability

The datasets presented in this study can be found in online repositories. The names of the repository/repositories and accession number(s) can be found below: https://github.com/susiejojo/distributed_GPU_multiagent_trajopt/.
